# Integrative Taxonomy of the *Caryospora simplex* Species Complex (Apicomplexa: Eimeriidae) From Vipers (Squamata: Viperidae), With Redescription and First Molecular Characterization of *Caryospora jararacae*

**DOI:** 10.1007/s11686-026-01411-0

**Published:** 2026-10-05

**Authors:** Arthur M. M. da Silva, Carlos Nei Ortúzar-Ferreira, Mariana de S. Oliveira, Leandro Dorna-Santos, Vitor de S. Ferreira, Viviane M. de Lima, Bruno P. Berto

**Affiliations:** 1 Programa de Pós-Graduação em Biologia Animal, Instituto de Ciências Biológicas e da Saúde, Universidade Federal Rural do Rio de Janeiro, BR- 465 km 7, Seropédica, Rio de Janeiro, 23897-000 Brazil; 2 Laboratório de Monitoramento Animal e Doenças Tropicais, Centro de Controle de Zoonoses de Miguel Pereira, Prefeitura Municipal de Miguel Pereira, Tv. Frederico Wangler 210, Vale da Pontresina, Miguel Pereira, Rio de Janeiro, 26900-000 Brazil; 3 Departamento de Biologia Animal, Instituto de Ciências Biológicas e da Saúde, Universidade Federal Rural do Rio de Janeiro, BR-465 km 7, Seropédica, Rio de Janeiro, 23897- 000 Brazil

**Keywords:** Coccidia, DNA barcoding, Multi-locus phylogeny, Evolutionary lineages, Host specificity, Neotropical Region

## Abstract

**Background:**

The genus *Caryospora* Léger, 1904 comprises facultatively heteroxenous coccidia that predominantly parasitize snakes, with seven nominal species recorded in vipers (Viperidae). In the Neotropical region, the Jararaca *Bothrops jararaca* (Wied-Neuwied, 1824) and the Common Lancehead *Bothrops atrox* (Linnaeus, 1758) were previously the only recorded viperid hosts for *Caryospora jararacae* (Carini, 1939). This study aimed to provide a morphological redescription and the first molecular characterization of *C. jararacae*, alongside a global taxonomic review of *Caryospora* spp. in viperids.

**Methods:**

Fecal samples from *B. jararaca* and Cascabel Rattlesnakes *Crotalus durissus* Linnaeus, 1758 were analyzed for oocyst morphology. Molecular characterization of *C. jararacae* was performed using a multilocus approach combining three mitochondrial loci and the nuclear 18S rDNA gene. Additionally, an integrative review synthesizing global data on geographic distribution, host specificity, and genotypic similarities of viperid *Caryospora* spp. was conducted.

**Results:**

*Caryospora jararacae* was identified and redescribed from *B. jararaca* and *C. durissus*. By reidentifying previous morphometric reports from *C. durissus* as *C. jararacae* and providing the first genetically confirmed records for both host species, this study establishes *C. jararacae* as the sole *Caryospora* species known from Neotropical vipers and only the second genotypically characterized *Caryospora* species in the family Viperidae worldwide. The integrative review revealed that five of the seven nominal species reported in vipers belong to the *Caryospora simplex* species complex, representing five morphologically indistinguishable geotypes distributed across different continents.

**Conclusions:**

Molecular evidence primarily supports a scenario of recent common ancestry and incipient speciation among geotypes of the *Caryospora simplex* species complex, wherein high genetic similarity reflects shared ancestral characters, rather than active ongoing genetic exchange. Despite their morphological indistinguishability, retention of the five nominal species within the complex is recommended, as they can be delimited through an integrative framework combining biogeographical, host, and molecular data.

**Supplementary Information:**

The online version contains supplementary material available at https://doi.org/10.1007/s11686-026-01411-0.

## Introduction

Coccidians belonging to the genus *Caryospora* Léger, 1904, are facultatively heteroxenous parasites that primarily infect snakes and other reptiles. Transmission to reptilian hosts occurs either via the fecal-oral route or through the predation of rodents (intermediate hosts) harboring caryocysts. This genus is characterized by monosporocystic, octozoic oocysts, and, consistent with the family Eimeriidae, by a sporocyst possessing a Stieda body as an excystation structure [1–3].

Upton et al. [4], Duszynski & Upton [5] and Duszynski [2, 6] provide the most comprehensive reviews of *Caryospora* to date. Since the study by Upton et al. [4], the number of *Caryospora* spp. recorded in snakes has increased from 18 to 56. Of these, only seven species have been recorded in vipers (Viperidae), including the type species, *Caryospora simplex* Léger, 1911. This species was originally described from the Asp Viper, *Vipera aspis* (Linnaeus, 1758), which is widely distributed across Europe [7–9].

Within the Neotropical region, Jararacas *Bothrops jararaca* (Wied-Neuwied, 1824) and Common Lanceheads *Bothrops atrox* (Linnaeus, 1758) were the first viperid hosts recorded for the genus *Caryospora*, specifically for *Caryospora jararacae* (Carini, 1939), which was originally described from *B. jararaca* and subsequently redescribed from *B. atrox* in Brazil [10, 11]. Jararacas and Cascabel Rattlesnakes *Crotalus durissus* Linnaeus, 1758 are two of the most medically significant venomous viperids in the densely populated eastern region of Brazil. Due to their high abundance and frequent encounters with humans, they are primary subjects for epidemiological and parasitological research [12–14].

In this context, the present study provides a morphological redescription and the first molecular characterization of *C. jararacae* from *B. jararaca* and *C. durissus*, utilizing three mitochondrial loci and one locus of the nuclear 18S rDNA gene. Furthermore, we provide a critical review of *Caryospora* spp. recorded from vipers worldwide. To re-evaluate their taxonomic status and evolutionary relationships through an integrative approach, we herein define the *‘Caryospora simplex* species complex’ to encompass *C. simplex* alongside morphologically closely related taxa (*C. jararacae*, *Caryospora bigenetica* Wacha et Christiansen, 1982, *Caryospora maculatus* Upton, Freed et Freed, 1992 and *Caryospora bothriechis* Seville, Asmundsson et Campbell, 2005). This synthesis integrates previously unexplored data on geographical distribution, host specificity, and genotypic similarities to propose taxonomic refinements for the genus.

## Materials and Methods

### Sample Collection

Fecal samples were obtained from five *B. jararaca* and five *C. durissus* rescued from urban environments in the municipality of Miguel Pereira, Rio de Janeiro state, Southeastern Brazil. These specimens were temporarily housed at the Laboratory of Animal Monitoring and Tropical Diseases (Laboratório de Monitoramento Animal e Doenças Tropicais – LABMADT), within the Zoonosis Control Sector of the Municipal Government of Miguel Pereira (Setor de Zoonoses da Prefeitura Municipal de Miguel Pereira – PMMP). One fresh fecal sample per specimen was collected and placed in a dedicated fecal collection vial with a 2.5% (w/v) aqueous potassium dichromate (K₂Cr₂O₇) solution. All animal captures, handling, and sampling procedures were approved by the Animal Ethics Committee (Comitê de Ética no Uso de Animais – CEUA) of the Federal Rural University of Rio de Janeiro (Universidade Federal Rural do Rio de Janeiro – UFRRJ) under process number 08/2026 (for detailed permit and ethics declarations, see the ‘Ethical Approval’ section).

### Morphological Analyses

The parasitological processing was conducted at the Laboratory of Biology of Coccidians (Laboratório de Biologia de Coccídios – LABICOC), UFRRJ. To facilitate sporulation, samples were maintained at 25 °C for seven days. Oocyst recovery was achieved through the Sheather’s sugar flotation method (1.20 specific gravity) [15]. Consistent with Bush et al. [16], prevalence was calculated based on the ratio of positive individuals to the total number of examined hosts. Morphological descriptions and measurements were performed using an Olympus BX microscope (Olympus Corporation, Tóquio, Japão) coupled with a Eurekam 5.0 camera (BEL Engineering, Monza, Itália), following standardized criteria [15, 17]. Visual outputs, including line drawings, photomicrographs, and composite figures, were prepared and adjusted using the CorelDRAW Graphics Suite 2020 package, which includes CorelDRAW and Corel PHOTO-PAINT (Corel Corporation, Ottawa, Canada). All measurements are provided in micrometres as ranges (mean in parentheses).

### Molecular Analyses

Single oocysts, previously analyzed morphologically and documented via photomicrography, were individually isolated, transferred into microtubes, and resuspended in 90 µL of phosphate-buffered saline (PBS), following the methodology established by Dolnik et al. [18]. To promote complete wall disruption and DNA release, samples were subjected to four freeze–thaw cycles (at − 4 °C and 96 °C, 5 min each). Following this step, 10 µL of proteinase K was added, and the mixture was incubated at 56 °C for approximately 2 h. DNA was then extracted using the Zymo Research Quick-DNA Kit (Zymo Research, Irvine, CA, USA), strictly adhering to the manufacturer’s protocol. PCR amplification targeted four distinct, non-overlapping loci across the mitochondrial and nuclear genomes. Specifically, the mitochondrial organization was assessed using the MAVCOXI locus (cytochrome c oxidase subunit 1, cox1; ~1600–2200 nt), the MARI locus (SSU and LSU rDNA fragments; ~2950–3750 nt), and the MACOIII locus (cytochrome c oxidase subunit 3, cox3; ~4200–4750 nt), as detailed by Ortúzar-Ferreira et al. [19]. Additionally, the nuclear 1NF locus, targeting the 18S rDNA gene (~ 800–1100 nt), was amplified following Genovez-Oliveira et al. [20]. Each 25 µL PCR reaction mixture contained 12.5 µL of GoTaq^®^ G2 Hot Start Colorless Master Mix (Promega, Madison, WI, USA), 3 µL of genomic DNA (< 1 µg), 0.25 µL of each primer (0.2 µM), and 9 µL of nuclease-free water. Thermal cycling conditions were applied as previously optimized by Ortúzar-Ferreira et al. [19] and Genovez-Oliveira et al. [20].

### DNA Sequence Analyses

Bidirectional Sanger sequencing of the PCR amplicons was conducted by Ludwig Biotechnology (Alvorada, RS, Brazil) using an ABI Prism 3500 Genetic Analyzer (Applied Biosystems, Foster City, CA, USA). The resulting sequence chromatograms were inspected and manually refined using Chromas v.2.6 (Technelysium Pty Ltd, Brisbane, Australia). To evaluate sequence similarity, data were queried against coccidian sequences in GenBank using the Basic Local Alignment Search Tool (BLASTn). Taxa were selected for inclusion only if they had available sequences overlapping across at least three of the four loci targeted in this study. Alignments for each locus were generated in MEGA v.10.2.6 using the ClustalW algorithm. A multi-locus concatenated dataset integrating sequences from the four targeted regions (MAVCOXI, MARI, MACOIII, and 1NF) was assembled for 20 taxa. *Choleoeimeria taggarti* (Amery-Gale, Devlin, Tatarczuch, Taggart, Schultz, Charles et Beveridge, 2018) served as the outgroup for all phylogenetic reconstructions. The best-fit nucleotide substitution model for the concatenated dataset was evaluated using the Model Selection tool in MEGA. The General Time Reversible model with gamma-distributed rate variation among sites and a proportion of invariable sites (GTR + G+I) was identified as the optimal model for the whole alignment under both the Bayesian Information Criterion (BIC) and corrected Akaike Information Criterion (AICc) (Supplementary Table S1). Pairwise genetic distances (p-distance) were estimated in MEGA using the uncorrected p-distance model incorporating both transitions and transversions, with pairwise deletion for missing data treatment (Supplementary Table S2). Phylogenetic reconstruction was executed under the GTR + G+I model for both Bayesian Inference (BI) via MrBayes v.3.2.7 [21] and Maximum Likelihood (ML) via MEGA [22]. For BI, Markov chain Monte Carlo (MCMC) simulations were run for 1,000,000 generations across two independent runs with four chains each, sampling topologies every 1,000th generation. A conservative burn-in fraction of 25% (250 samples) was discarded prior to constructing the majority-rule consensus tree. Chain convergence was confirmed by an Average Standard Deviation of Split Frequencies of 0.017 (ASDSF = 0.017) and an average Potential Scale Reduction Factor of 1.001 (PSRF = 1.001). The ML tree was inferred under identical model parameters (GTR + G+I, 95% partial deletion cutoff), with branch support evaluated through 1,000 bootstrap pseudo-replicates. Final tree topologies were visualized and formatted using FigTree v.1.4.4 [23].

## Results

### Prevalence and Description

Coccidian oocysts were detected in three out of five (60%) *B. jararaca* and four out of five (80%) *C. durissus* specimens housed at LABMADT (PMMP). All analyzed oocysts were morphologically identified to the genus *Caryospora*. Based on the qualitative and quantitative taxonomic characters of the oocysts examined, the specimens align with the *C. simplex* species complex (Table 1). The integration of the morphology with biogeographic data, host specificity, and molecular characterization allowed for the specific identification as *C. jararacae*. A standardized redescription of *C. jararacae* follows, alongside a comparative analysis and a critical evaluation of the validity of related species.

**Table 1:** Taxonomic comparison of *Caryospora* spp. from vipers (Squamata: Viperidae) based on morphological and morphometric characters

Coccidian species		Type host	Other hosts	Distribution (continents)	Reference
***Caryospora simplex*** **species complex**	*Caryospora simplex* (Léger, 1911)	*Vipera aspis* (Linnaeus, 1758)	*Daboia palaestinae* (Werner, 1938); *Daboia russelii* (Shaw et Nodder, 1797); *Montivipera xanthina* (Gray, 1849); *Vipera ammodytes* (Linnaeus, 1758); *Vipera berus* (Linnaeus, 1758); *Vipera kaznakovi* Nikolsky, 1909	Europe; Asia	Léger [8]; Upton et al. [4, 24]; Upton and Barnard [25]; Matuschka [26]; Wilber et al. [27]; Modry et al. [28]
	***Caryospora jararacae*** **(Carini**,** 1939)**	***Bothrops jararaca*** **(Wied-Neuwied**,** 1824)**	***Bothrops atrox*** **(Linnaeus**,** 1758);** ***Bothrops jararacussu*** **Lacerda**,** 1884;** ***Bothrops moojeni*** **Hoge**,** 1966;** ***Crotalus durissus*** **Linnaeus**,** 1758**	**South America**	Carini [10]
					Lainson et al. [11]
					Viana et al. [29]^1^
					**present study**
	*Caryospora bigenetica* Wacha et Christiansen, 1982	*Crotalus horridus* Linnaeus, 1758	*Agkistrodon contortrix* (Linnaeus, 1766); *Crotalus adamanteus* Beauvois, 1799; *Crotalus atrox* Baird et Girard, 1853; *Sistrurus catenatus* (Rafinesque, 1818)	North America	Wacha & Christiansen [30]; Upton et al. [4]; Upton & Barnard [31]; Koudela [32]
	*Caryospora maculatus* Upton, Freed et Freed, 1992	*Causus maculatus* (Hallowell, 1842)	not reported	Africa	Upton et al. [33]
	*Caryospora bothriechis* Seville, Asmundsson et Campbell, 2005	*Bothriechis aurifer* (Salvin, 1860)	not reported	Central America	Seville et al. [36]
*Caryospora matatu* Modrý, Šlapeta et Koudela, 2002		*Atheris ceratophora* Werner, 1895	not reported	Africa	Modrý et al. [34]
*Caryospora saudiarabiensis* (Modrý, Koudela, Al-Oran et Amr, 1999)^2^		*Echis carinatus* (Schneider, 1801)	*Echis coloratus* Günther, 1878	Asia	Alyousif et al. [35]; Duszynski & Upton [5]

Coccidian species		Oocysts							
		Shape	L^3^ (µm)	W^4^ (µm)	L/W^5^	Wall (µm)	Micropyle	Polar granule (µm)	Residuum
***Caryospora simplex*** **species complex**	*Caryospora simplex* (Léger, 1911)	subspheroidal	13.5–16 (14.9)	13.5–16 (14.9)	(1.0)	lightly pitted; (1.4)	absent	1–2; attached to the oocyst wall	absent
	***Caryospora jararacae*** **(Carini**,** 1939)**	subspheroidal	13–14	13–14	not reported	thick	absent	absent	absent
		subspheroidal	12–14 (13)	12–14 (13)	1.0–1.1 (1.0)	smooth; (0.7)	absent	1; subspheroidal; (1.0); adhered to oocyst wall	absent
		subspheroidal	12–14 (13)	12–14 (13)	(1.0)	outer surface slightly mammillated; striations visible in cross-section; (1.0)	absent	1; fixed in the internal wall	absent
		**subspheroidal**	**13–16 (14.1)**	**12–16 (13.8)**	**1.0–1.1 (1.02)**	**smooth to finely rough; (1.0)**	**absent**	**1; in 52.5% of oocysts; subspheroidal; (1.6 × 1.3); adhered to oocyst wall**	**absent**
	*Caryospora bigenetica* Wacha et Christiansen, 1982	subspheroidal	11–15 (13.2)	11–15 (13.2)	(1.0)	smooth to mamillated; (1.0)	absent	1; attached to the oocyst wall	absent
	*Caryospora maculatus* Upton, Freed et Freed, 1992	subspheroidal	11–13 (12.1)	11–13 (12.1)	(1.0)	smooth; (1.0)	absent	1; in 12.5% of oocysts	absent
	*Caryospora bothriechis* Seville, Asmundsson et Campbell, 2005	subspheroidal	12–14 (12.7)	12–13 (12.5)	(1.0)	smooth; (0.8)	absent	1; refractile; attached to the oocyst wall	absent
*Caryospora matatu* Modrý, Šlapeta et Koudela, 2002		subspheroidal	16–23 (19.8)	16–23 (19.8)	(1.0)	brownish; distinctly pitted; (1.5)	absent	1; subspheroidal; (1.0); close to the oocyst wall	2–5 small granules in some oocysts
*Caryospora saudiarabiensis* (Modrý, Koudela, Al-Oran et Amr, 1999)^2^		subspheroidal	40–46 (42.8)	40–44 (41.2)	1.0–1.1 (1.0)	smooth; (1.9)	absent	1; subspheroidal; sometimes absent	absent

Coccidian species		Sporocysts						
		Shape	L^1^ (µm)	W^2^ (µm)	L/W^3^	Stieda body (µm)	Sub-Stieda body (µm)	Residuum
***Caryospora simplex*** **species complex**	*Caryospora simplex* (Léger, 1911)	ovoidal	10–13 (11.6)	8–9.5 (8.9)	1.3	present; (1 × 2.5)	present; (1.5 × 2.5)	numerous granules of various sizes
	***Caryospora jararacae*** **(Carini**,** 1939)**	ovoidal	9–10	7–8	not reported	present	not reported	formed by some granulations
		broadly ellipsoidal	9–11 (10.0)	7–9 (8.7)	1.1–1.3 (1.1)	stopper-like	present	consisting of ~ 20 subspheroid granules
		ovoidal or pyriform	9–11 (10.0)	8–9 (8.0)	(1.3)	discoid	present	formed by a group of spheroid bodies
		**broadly ellipsoidal**	**9–13 (11.4)**	**8–10 (8.5)**	**1.2–1.5 (1.33)**	**flattened to knob-like; (0.7 × 1.8)**	**rounded to trapezoidal; (1.2 × 2.4)**	**consisting of large spherules**
	*Caryospora bigenetica* Wacha et Christiansen, 1982	ellipsoidal, ovoidal, or pyriform	8–11.5 (10.1)	6.5–9 (7.7)	1.3	discoidal of uniform thickness; (1.0 × 1.5)	same width as Stieda body	cluster of several spheroidal bodies
	*Caryospora maculatus* Upton, Freed et Freed, 1992	ovoidal	9.5–11 (10.1)	7.5–8.5 (7.9)	1.2–1.4 (1.3)	present	small and inconspicuous	cluster of granules with a central globule
	*Caryospora bothriechis* Seville, Asmundsson et Campbell, 2005	ovoidal	8–10 (9.0)	7–8 (7.5)	not reported	faint, clear	absent	few disbursed small granules
*Caryospora matatu* Modrý, Šlapeta et Koudela, 2002		ellipsoidal	12.5–17 (15.6)	8–12 (10.1)	1.4–1.7 (1.5)	domelike; (1 × 2.5)	present; (2 × 4); indistinguishable in some sporocysts	numerous small granules scattered
*Caryospora saudiarabiensis* (Modrý, Koudela, Al-Oran et Amr, 1999)^2^		broadly ellipsoidal	21–23 (22.1)	15.5–17 (16.8)	1.2–1.5 (1.3)	domelike; (1.5 × 3.5)	(4.0 × 4.5)	many granules in a compact mass

^1^Misidentified as *Caryospora bigenetica* in Viana et al. [29]

^2^Established by Duszynski & Upton [5] to accommodate specimens from *Echis* spp. previously misidentified as *Caryospora maxima* Modrý, Koudela, Al-Oran and Amr, 1999 by Alyousif et al. [35]

^3^Length

^4^Width

^5^Ratio of length to width

### *Caryospora jararacae* (Carini, 1939) (Figs. 1 and 2)

Subclass: Coccidia Leuckart, 1879

Family: Eimeriidae Minchin, 1903

Genus: *Caryospora* Léger, 1904

Oocyst (*n* = 40) subspheroidal, 13–16 × 12–16 (14.1 × 13.8); L/W ratio 1.0–1.1 (1.02). Wall bi-layered, 0.6–1.3 (1.0) thick; outer layer smooth to finely rough, comprising approximately two-thirds of total thickness. Micropyle and oocyst residuum absent. Polar granule present in 52.5% (21/40) of oocysts, usually adhered to the oocyst wall, subspheroidal, 1.4–1.6 × 1.1–1.5 (1.6 × 1.3). Sporocyst (*n* = 37) broadly ellipsoidal, 9–13 × 8–10 (11.4 × 8.5); L/W ratio 1.2–1.5 (1.33). Stieda body present, flattened to knob-like, 0.6–0.8 × 1.7–2.0 (0.7 × 1.8). Sub-Stieda body present, rounded to trapezoidal, 1.1–1.3 × 2.3–2.6 (1.2 × 2.4). Para-Stieda body absent. Sporocyst residuum present, consisting of large spherules scattered among the sporozoites. Sporozoites vermiform, each with anterior and posterior refractile bodies and a central nucleus.

**Fig. 1:**
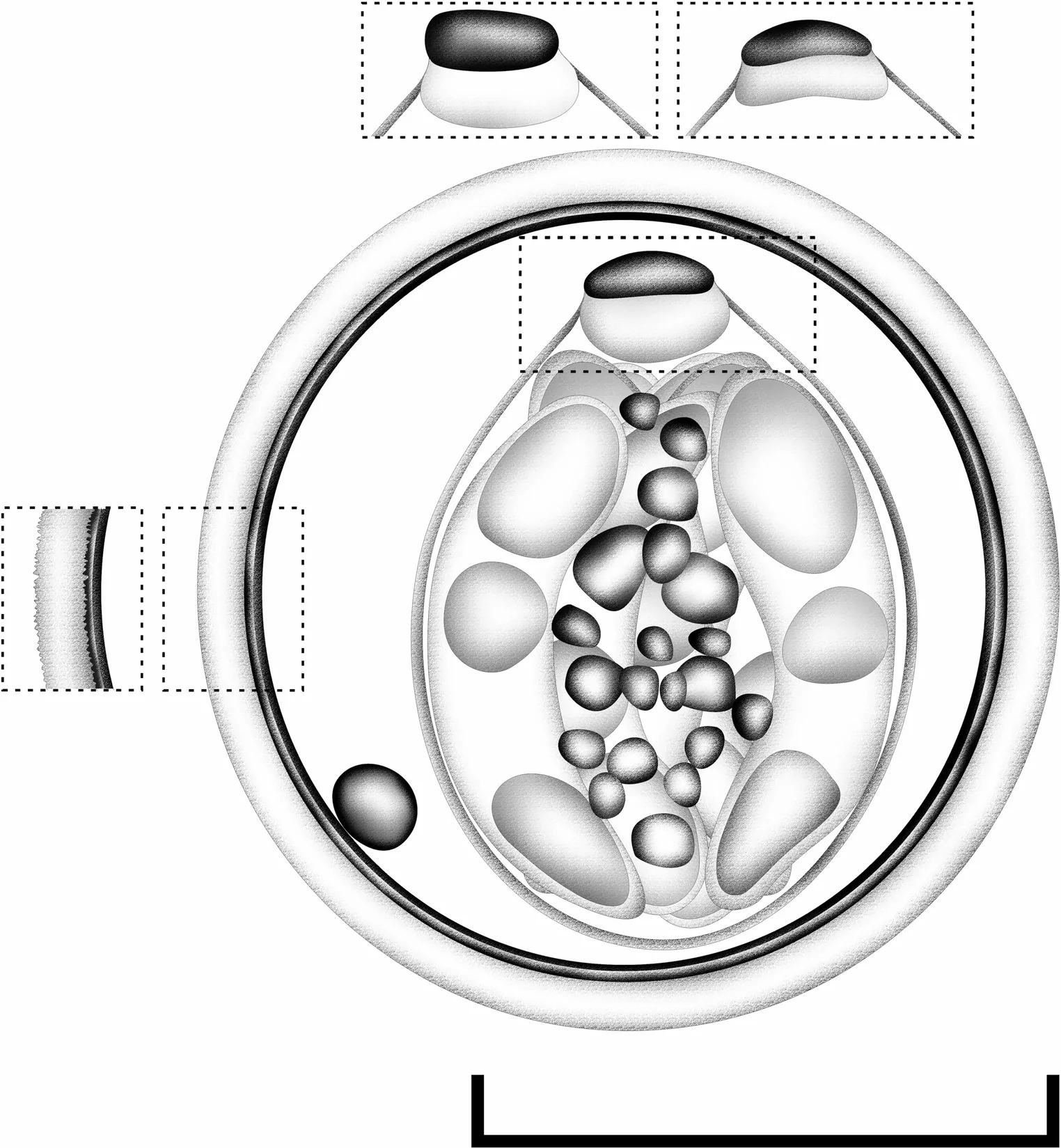
Composite line drawing of the sporulated oocyst of *Caryospora jararacae* from Jararacas *Bothrops jararaca* and Cascabel Rattlesnakes *Crotalus durissus* in Southeastern Brazil. Dashed lines highlight the observed intraspecific variations in the oocyst wall and the shapes of the Stieda and sub-Stieda bodies. Scale bar = 10 μm

**Fig. 2:**
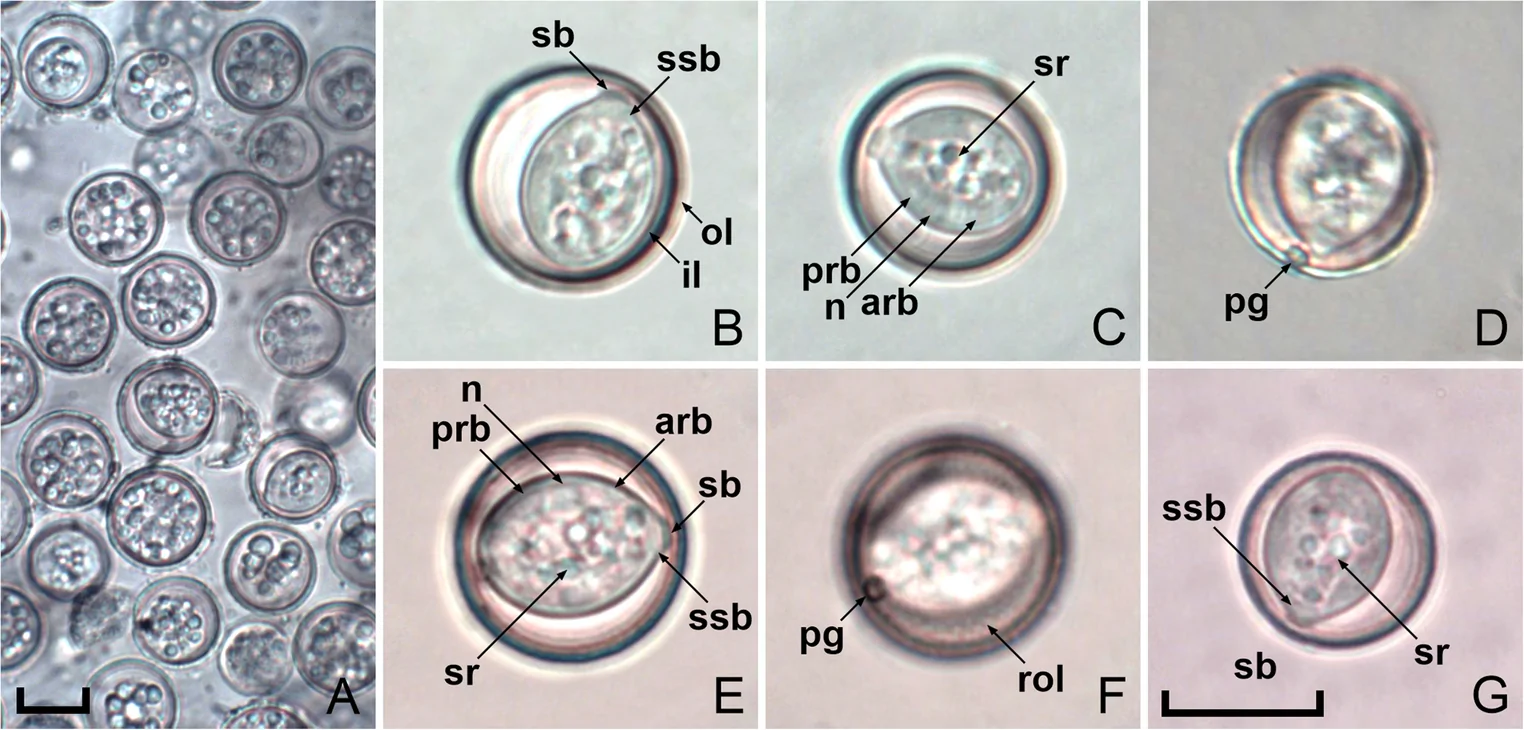
Photomicrographs of oocysts of *Caryospora jararacae*. **A** Low-magnification view of a fecal sample with high oocyst density from a Jararaca *Bothrops jararaca*, showing oocysts at different stages of sporogony. **B**–**G** Sporulated oocysts at higher magnification, highlighting key morphological taxonomic characters, from *B. jararaca* (**B**–**D**) and Cascabel Rattlesnakes *Crotalus durissus* (**E**–**G**). Abbreviations: anterior (arb) and posterior (prb) refractile bodies; nucleus (n); outer (ol), finely rough outer (rol), and inner (il) layers of the oocyst wall; polar granule (pg); sporocyst residuum (sr); Stieda body (sb); sub-Stieda body (ssb). Scale bars: A and B–G = 10 μm

#### Diagnosis

Following the guidelines of Duszynski & Wilber [15] and Berto et al. [17], the oocysts obtained from *B. jararaca* and *C. durissus* in this study were compared with those previously recorded from hosts within the same family, namely Viperidae (Table 1). To date, seven nominal *Caryospora* spp. have been reported for this host family, five of which exhibit mostly equivalent characteristics. These five species were herein grouped into the *C. simplex* species complex, as *C. simplex* is the oldest described within this morphotype, in addition to being the type-species of the genus *Caryospora*.

Minor quantitative and qualitative differences among these species make a delimitation based purely on morphological and/or morphometric taxonomic characters unreliable. The reported absence of a sub-Stieda body in *C. bothriechis* is the only feature that would clearly distinguish it from others in the complex; however, this absence is questionable upon inspection of the photomicrographs (photosyntypes) provided by Seville et al. [36].

The oocysts in the present study were equivalent in all taxonomic characters when compared to the species of the *C. simplex* species complex; therefore, they were identified as *C. jararacae*, fundamentally based on their geographic distribution. Conversely, *Caryospora matatu* Modrý, Šlapeta et Koudela, 2002 and *Caryospora saudiarabiensis* (Modrý, Koudela, Al-Oran et Amr, 1999) can be easily distinguished from the current specimens of *C. jararacae* by size, as well as features such as the presence of an oocyst residuum in *C. matatu* and a compact sporocyst residuum in *C. saudiarabiensis* (Table 1).

#### Taxonomic Summary

- Synonymies: *Caryospora jaracae* Carini, 1939 (*lapsus calami*); *Caryospora bigenetica sensu* Viana et al. [29].
- Type host: *Bothrops jararaca* (Wied-Neuwied, 1824), Jararaca (Reptilia: Squamata: Viperidae) [10].
- Type locality: São Paulo, Brazil [10].
- Other hosts: *Bothrops atrox* (Linnaeus, 1758), Common Lancehead; *Bothrops jararacussu* Lacerda, 1884, Jararacussu; *Bothrops moojeni* Hoge, 1966, Brazilian Lancehead; *Crotalus durissus* Linnaeus, 1758, Cascabel Rattlesnake [11, 29].
- Other localities: Amazonas, Brazil; Mato Grosso do Sul, Brazil [11, 29].
- Type material: Not stated in the original description by Carini [10].
- Neotype: Deposited in the Parasitological Collection of the Instituto Evandro Chagas, Belém, Pará, Brazil, under repository number FN25 (designated by Lainson et al. [11]).
- Voucher specimens (present study): Photomicrographs, line drawings, and oocysts in 2.5% K_2_Cr_2_O_7_ solution are deposited in the Parasitology Collection of the LABICOC at UFRRJ, Seropédica, Brazil, under repository number 142/2026. Digital records are available at http://r1.ufrrj.br/labicoc/colecao.html.
- Representative DNA sequences: Multi-locus DNA amplification of the MAVCOXI, MARI, MACOIII, and 1NF loci yielded clear bands at approximately 650 bp, 800 bp, 630 bp, and 440 bp, respectively. Representative sequences are deposited in GenBank under the following accession numbers: PZ316317 and PZ316318 (MAVCOXI from *B. jararaca* and *C. durissus*); PZ339031 and PZ339032 (MARI from *B. jararaca* and *C. durissus*); PZ316319 and PZ316320 (MACOIII from *B. jararaca* and *C. durissus*); and PZ291766 and PZ291767 (1NF from *B. jararaca* and *C. durissus*).
- ZooBank registration: The updated taxonomic data and the supplemental redescription provided in this study were registered in ZooBank, as per the requirements of the International Code of Zoological Nomenclature [37]. The Life Science Identifier (LSID) for this publication is urn: lsid: zoobank.org: pub:4BA5D422-CB4B-41D7-98D7-5090D7749E14 and the LSID for the registered taxon *Caryospora jararacae* (Carini, 1939) is urn: lsid: zoobank.org: act:645D0C26-D0BE-4C26-8749-49B8A204651D.
- Site of infection: Unknown; oocysts were recovered from feces.
- Prevalence: The overall prevalence was 70% (7/10). Specifically, the prevalence was 60% (3/5) for *B. jararaca* and 80% (4/5) for *C. durissus*.

#### Molecular and phylogenetic analyses

DNA sequences obtained from the four non-overlapping mitochondrial (MAVCOXI, MARI, and MACOIII) and nuclear (1NF) loci were 100% identical among samples from the same host species. When comparing samples from different hosts (*B. jararaca* vs. *C. durissus*), sequences were also 100% identical, with the sole exception of a minor 0.4% difference (745/748 bp) in the MARI locus, corresponding to a 3-nucleotide substitution.

When compared with Eimeriidae sequences available in GenBank, *C. jararacae* sequences from the present study showed the highest identity with the complete mitochondrial genome and nuclear 18S rDNA sequences of the only *Caryospora* sp. from vipers deposited to date: *Caryospora bigenetica* obtained from the Massasauga *Sistrurus catenatus* (Rafinesque, 1818) [38, 39]. High identity scores with this species were observed across all loci: 1NF (100%; 365/365 bp), MARI (99.3%; 761/766 bp), MAVCOXI (99.0%; 613/619 bp), and MACOIII (97.7%; 542/555 bp). The second most similar species was *Isospora amphiboluri* McAllister, Upton, Jacobson et Kopit, 1995 from the Central Bearded Dragon *Pogona vitticeps* (Ahl, 1926) [40–42]; however, similarities were significantly lower, at 93% for mitochondrial loci and 99% for the nuclear 18S gene.

Other *Caryospora* spp. from non-viperid hosts deposited in GenBank were only partially sequenced for the loci analyzed in the present study. An undescribed *Caryospora* sp. isolated from the Greater Green Snake *Ptyas major* (Günther, 1858) [43] had its complete mitochondrial genome sequenced, exhibiting sequence similarities of 90% for MAVCOXI and MACOIII, and 95% for MARI; however, no nuclear 18S rRNA sequence was available for this isolate. Two additional undescribed *Caryospora* spp. possess partial sequences restricted to the cox1 gene: a *Caryospora* sp. from the Short-bearded Anole *Anolis chamaeleonides* Duméril et Bibron, 1837 [44], which shared 93% similarity at the MAVCOXI locus, and a *Caryospora*-like isolate from the Magpie-lark *Grallina cyanoleuca* (Latham, 1801) [45], which exhibited 91% similarity at the same locus. The detection from *G. cyanoleuca* may represent a case of pseudoparasitism (spurious passage), resulting from the ingestion and passage of oocysts through the digestive tract of an avian predator that consumed an infected reptile host [46]. Regarding the nuclear 18S gene, *Caryospora ernsti* Upton, Current et Barnard, 1984 from the Green Anole *Anolis carolinensis* (Voigt, 1832) [42], a *Caryospora* sp. from the Indian Cobra *Naja naja* (Linnaeus, 1758) [47], the *Caryospora*-like isolate from *G. cyanoleuca* [45] and *Caryospora cheloniae* Leibovitz, Rebell & Boucher, 1978 from the Green Turtle *Chelonia mydas* (Linnaeus, 1758) [48], all displayed high sequence similarity values ranging from 97% to 99%. Notably, the generic assignment of the severely pathogenic sea turtle coccidian (*C. cheloniae*) within *Caryospora* has been questioned [3], highlighting the need for further taxonomic studies to validate or reclassify this taxon.

The concatenated dataset derived from these four loci comprised 2,438 base pairs. As expected, *C. bigenetica* exhibited the highest sequence similarity at 99.0% (2,414/2,438 bp), followed by *I. amphiboluri* at 95.2% (2,321/2,438 bp). In the pairwise genetic distance matrix (Supplementary Table S2), *C. bigenetica* (98.9% identity) and *I. amphiboluri* (94.7% identity), both originating from reptilian hosts, were confirmed as the most closely related coccidians to the *C. jararacae* isolates evaluated in this study. These were more distantly followed by avian *Isospora* spp. and *Cyclospora cayetanensis* Ortega, Gilman et Sterling, 1994, which shared identities ranging from 92.8% to 93.6%. Furthermore, *Caryospora* sp. from *P. major* displayed an identity of 92.2%.

**Fig. 3:**
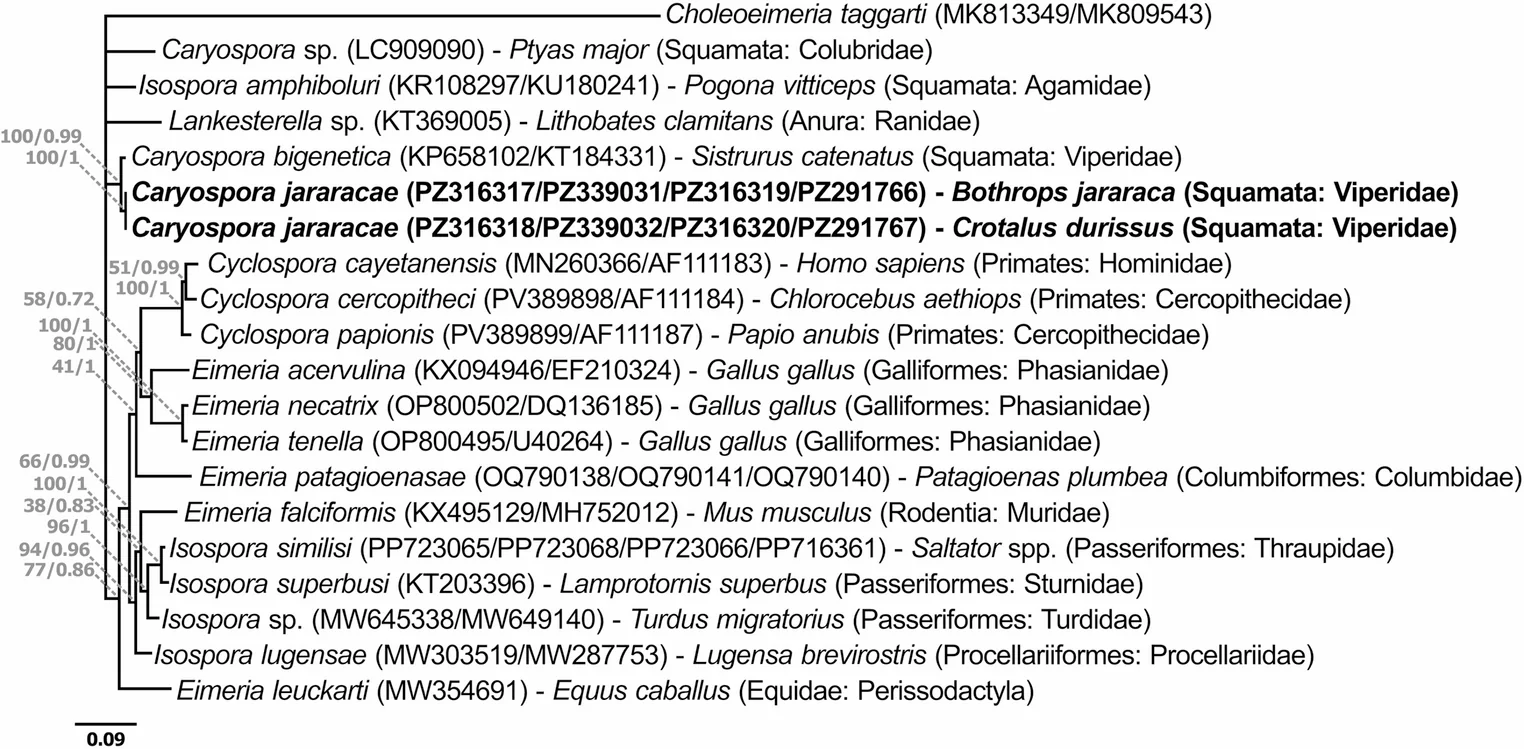
Phylogenetic relationships of *Caryospora jararacae* from Jararacas *Bothrops jararaca* and Cascabel Rattlesnakes *Crotalus durissus*, inferred by Bayesian inference based on concatenated sequences of three mitochondrial loci and one nuclear 18S rDNA locus. Branch lengths represent the mean posterior estimates of evolutionary distances (scale bar = 0.09 substitutions per site). Node labels indicate maximum likelihood bootstrap support values (left) and Bayesian posterior probabilities (right). The tree was rooted using *Choleoeimeria taggarti* as the outgroup

In the phylogenetic analysis based on the concatenated multi-locus dataset (Fig. 3), *C. jararacae* (isolated from *B. jararaca* and *C. durissus*) formed a highly supported monophyletic clade with *C. bigenetica* (posterior probability = 0.99; bootstrap = 100%). In the Bayesian consensus topology, this viperid-parasitizing *Caryospora* clade was positioned within a basal polytomy alongside other early-diverging reptilian lineages, including *I. amphiboluri*, *Lankesterella* sp., and *Caryospora* sp. from *P. major*, and the main lineage comprising other eimeriid genera.

## Discussion

The genus *Caryospora*, originally established within the Eimeriidae, underwent significant systematic reorganization after molecular and morphological evidence revealed that species parasitizing raptors belong to the family Sarcocystidae, now assigned to the genus *Eumonospora* Allen, 1933 [2, 49]. Beyond the absence of a Stieda body in *Eumonospora* spp., a remarkable biological distinction underscores the high host specificity of *Caryospora* toward snakes. While *Caryospora* spp. induce cyst formation in the oral musculature of rodents, impairing their feeding and agility to facilitate predation by snakes, *Eumonospora* spp. develop cysts primarily in the dermal connective tissue. This strategy preserves the mobility of the prey, ensuring the visual stimulus required for raptor predation [4, 49, 50]. Such specialized manipulative behavior in the intermediate host highlights the deep evolutionary commitment of *Caryospora* to the snake-prey trophic link.

**Fig. 4:**
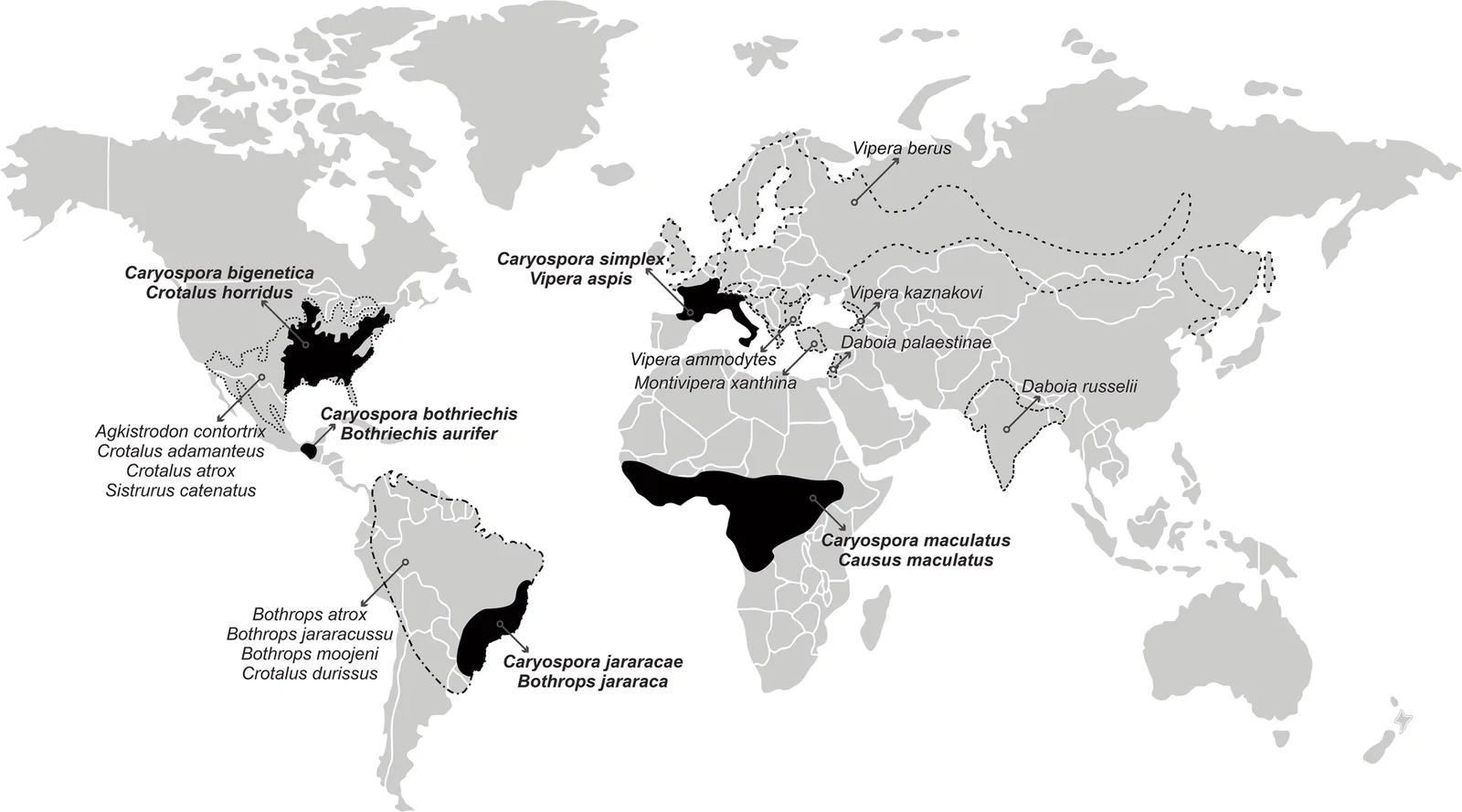
Global geographic distribution of the five *Caryospora* spp. belonging to the *Caryospora simplex* species complex, based on their type hosts and additional reported hosts. Solid black areas represent the ranges of the respective type hosts. Dotted lines delineate the distributions of additional hosts of *Caryospora bigenetica*. Dashed lines indicate the distributions of additional hosts of *Caryospora simplex*. Dash–dot lines outline the distributions of additional hosts of *Caryospora jararacae*. Host distribution data were adapted from the IUCN Red List of Threatened Species [9]. The base world map was adapted from Vecteezy (https://www.vecteezy.com)

These findings indicate that *Caryospora* spp. described from snakes exhibit a more robust taxonomic foundation compared to the few species reported from birds, lizards, and turtles, primarily due to their specialized co-evolutionary host relationship and more comprehensive morphological descriptions [2, 4–6]. *Caryospora simplex*, the type species established in the early 20th century, has been extensively studied through experimental infections and reports across various Eurasian viperid hosts. Subsequently, species such as *C. jararacae*, *C. bigenetica*, *C. maculatus*, and *C. bothriechis* were described from viperids across different continents; however, these descriptions often lack consistent morphometric delimitation, relying heavily on geographic distribution for identification (Fig. 4; Table 1).

This study confirms such taxonomic ambiguity, as the expanded morphometric ranges obtained for oocysts and sporocysts of *C. jararacae* overlapped with those of all aforementioned species. Furthermore, minor qualitative features previously used to distinguish these five *Caryospora* spp. were observed as intraspecific variations within the analyzed samples. For instance, the observation of an oocyst wall surface that is not absolutely smooth, but lightly pitted, mamillated, or finely rough in some oocysts, is a feature previously ascribed to *C. simplex* and *C. bigenetica*. Likewise, the presence of a polar granule in only a fraction of the oocysts, a characteristic described for *C. maculatus*, was also observed in the current material, suggesting it is an inconsistent feature for diagnosis. Similarly, the single-layered oocyst wall described for *C. jararacae* and *C. simplex* appears to be a misinterpretation. While Viana et al. [29] identified *C. bigenetica* from *B. moojeni*, *B. jararacussu*, and *C. durissus* in Brazil based on this wall distinction, these reports were re-evaluated as *C. jararacae* in the current study (Table 1).

Beyond the low biogeographic probability of *C. bigenetica* dispersing from North American to Brazilian viperids (Fig. 4), ultrastructural studies by Belli et al. [51] and Mai et al. [52] have convincingly demonstrated that eimeriid oocyst walls consistently comprise two distinct layers. Although historical descriptions and even the guidelines by Duszynski & Wilber [15] refer to single-layered walls, these should be interpreted as two thin or fused layers [17, 51, 52]. Consequently, based on these morphological and morphometric equivalencies, these five *Caryospora* spp. from viperids worldwide are herein considered part of the *C. simplex* species complex.

Under a strictly morphological perspective, the five species comprising the *C. simplex* species complex would be considered synonyms. According to the International Code of Zoological Nomenclature, *C. simplex* would hold nomenclatural priority as the senior synonym, while the remaining four names would be invalidated as junior synonyms [53]. However, biogeographic and host specificity approaches provide a coherent framework for delimiting these species.

As demonstrated (Fig. 4), these five species are geographically segregated based on the distribution of their type-hosts and subsequent records. From a purely biogeographic standpoint, each lineage (geotype or vicariant population) is distributed across a specific continent, with Oceania remaining the only region without a described species or report for this complex. The distinction of these geotypes by biogeographic realms would be theoretically better founded, as these divisions consider large areas of the biosphere with unique fauna and flora, delimited by natural barriers and evolutionary history, such as continental drift. However, a strictly realm-based approach would fail to fully distinguish these geotypes, given that two species within the *C. simplex* species complex (*C. jararacae* and *C. bothriechis*) are distributed within the Neotropical region, while *C. simplex* itself spans two different zoogeographical realms: the Palearctic and the Oriental. Therefore, for didactic and clarifying purposes, these geotypes/species were separated based on classical geographical (continental) delimitation.

Regarding host specificity, it is generally assumed that the genus *Caryospora*, similar to other eimeriids, exhibits a parasitic specificity restricted at most to the host family level [15]. Nonetheless, some Eimeriidae species display narrower specificities at the subfamily or genus levels [54, 55]. Furthermore, host specificity can be highly variable and flexible, often shaped by adaptation and host-switching processes rather than rigid parasite-host coevolution [54, 56, 57]. For *C. simplex*, the lack of strict host specificity has been evident since the work of Upton & Barnard [25], who demonstrated that *C. simplex*, originally described from *V. aspis* in Europe, could experimentally infect and complete its life cycle in *Montivipera xanthina* (Gray, 1849) from the Middle East. Moreover, reports of *C. simplex* in other Eurasian hosts, such as *Daboia* spp. and *Vipera* spp., highlight the broad range of susceptible hosts within the family Viperidae. The other species within the *C. simplex* species complex corroborate this observation, as they involve hosts from different genera [2]. This is further supported by the current study, in which snakes from both *Crotalus* and *Bothrops* genera were found to shed oocysts of the same *Caryospora* sp.

In this context, host specificity alone does not appear sufficient to support the delimitation of *Caryospora simplex* species complex geotypes into distinct species. Conversely, an integrative taxonomic approach incorporating the spatio-temporal evolution of both hosts and geotypes provides a more robust framework for their delimitation. Overall, these five *Caryospora* geotypes distributed across different continents likely share a relatively recent common ancestor. Their morphological indistinguishability strongly suggests an incipient speciation process, driven by insufficient evolutionary time for structural separation and diversification among these geotypes. Under this scenario of recent divergence, the high genetic similarity could primarily reflect the retention of ancestral genetic characters without requiring ongoing genetic exchange. Alternatively, as a secondary hypothesis, it could be speculated that geographic barriers between certain geotypes are not fully impermeable, where potential gene flow might be facilitated by broad-ranged or migratory hosts acting as bridges in regions of sympatry [58, 59].

In this context, among the 124 viperid species of the New World, none are distributed simultaneously across both North and South America; however, 14 species occur in both North and Mesoamerica, and six are shared between Mesoamerica and South America. This higher species overlap between North and Mesoamerica suggests that the traditional boundary between the Nearctic and Neotropical regions (roughly between North and Central America) represents a relatively permeable transition for Viperidae, whereas a sharper biogeographical turnover occurs between Mesoamerica and South America. Regarding the Old World, of the 188 recorded viperid species, only six occur in both Eurasia and Africa, thus potentially facilitating the dispersal and transmission of *Caryospora* geotypes between these continents [9].

Regarding rodents, families such as Cricetidae, Muridae, Heteromyidae, and Nesomyidae are the most relevant prey for viperid snakes [60]. Of the 900 species from these families reported in Eurasia and Africa, only 20 occur in both regions. Among the 631 rodent species of these same families in the New World, none are distributed across both North and South America [9].

Based on these biogeographic data, while natural continuous dispersal of *Caryospora* spp. between North and South America is limited by the lack of shared host species, regional range expansions, such as that documented for *C. durissus* in Brazil, demonstrate the dynamic nature of these host-parasite systems. *Crotalus durissus* is typically associated with open, dry vegetation types such as the Cerrado, Caatinga, and Chaco; however, its expansion into the Atlantic Forest was likely facilitated by major flood events during the mid-20th century, allowing it to establish populations in deforested areas converted into pastures [61]. Furthermore, European colonization and transatlantic maritime traffic introduced synanthropic murids (*Rattus* spp. and *Mus musculus* Linnaeus, 1758) across the Americas. These rodents initially established along the coast and subsequently expanded inland alongside human settlements, becoming primary prey for synanthropic vipers [62]. Notably, these transformed peridomiciliary environments mirror the exact conditions where the viperid host specimens in the present study were rescued in the municipality of Miguel Pereira, Rio de Janeiro state, Southeastern Brazil. In this context, the introduction and proliferation of these synanthropic rodent species may have facilitated the historical dispersal, establishment, and transmission dynamics of *C. simplex* species complex geotypes in the Americas.

In any case, ongoing natural dispersal among the different geotypes of the *C. simplex* species complex across continents is constrained by vast oceanic barriers. Nevertheless, the role of anthropogenic dispersal through the illegal wildlife trade or legal animal commerce must be highlighted. This possibility is so significant that most descriptions and reports of *C. simplex* from Eurasian snakes were actually conducted in North America, using specimens held in breeding facilities and zoos [4, 24, 25, 27]. Considering the potential for environmental contamination with oocysts near such facilities, or transmission to local rodents preyed upon by native snakes, anthropogenic dispersal could theoretically bridge these geotypes and influence their evolutionary trajectories. This scenario underscores how globalization and the wildlife trade might impact the genetic architecture of parasites that historically evolved under geographic isolation, highlighting the need for ongoing genomic surveillance to monitor the potential homogenization of *C. simplex* species complex geotypes due to human activities [59].

These approaches and assumptions are corroborated by the molecular results of the present study. Although this is only the second *Caryospora* sp. from vipers to be characterized via molecular sequencing, the comparison between *C. bigenetica* and *C. jararacae* revealed a subtle yet distinct genotypic difference between the North American (*C. bigenetica*) and South American (*C. jararacae*) geotypes of the *C. simplex* species complex. Differences were primarily observed in mitochondrial genes, which, due to their higher mutation rates, are indicative of more recent evolutionary events and are therefore more suitable for species delimitation. The absolute identity observed within the analyzed ~ 365 bp 18S rRNA gene fragment (1NF locus) was expected, as it is a highly conserved marker more appropriate for phylogenetic positioning at higher taxonomic levels [3].

While natural dispersal or human transport could theoretically facilitate secondary contact, the minimal genetic divergence between North and South American *Caryospora* geotypes primarily supports a scenario of recent common ancestry. That is, rather than active gene flow, this high sequence identity is most consistently explained by the retention of shared ancestral traits following geographical isolation, where insufficient evolutionary time has elapsed for substantial differentiation.

Finally, there are no isolated taxonomic characters, whether morphological, biological, or ecological, that allow for a full distinction among the five geotypes of the *C. simplex* species complex. However, through an integrative taxonomic approach combining biogeographic and molecular data, a well-founded delimitation becomes possible.

Under a more traditional taxonomic assessment, these minimal differences could be relegated to the rank of subspecies [53]. In this case, all would be regarded as *C. simplex* under trinomial nomenclature, such as: *Caryospora simplex simplex*, *Caryospora simplex jararacae*, *Caryospora simplex bigenetica*, *Caryospora simplex maculatus*, and *Caryospora simplex bothriechis*. Nevertheless, considering the importance of nomenclatural stability and the practical challenges of adopting a widespread taxonomic revision, combined with the fact that the use of subspecies is exceedingly rare within the Eimeriidae, this possibility is not recommended in the present study.

Another possibility would be the synonymization of these five species under *C. simplex*, differentiating the geotypes as nominal genotypes, similar to the framework established for *Toxoplasma gondii* (Nicolle et Manceaux, 1908) [63]. However, such differentiation relies fundamentally on molecular analysis, currently available only for the North and South American geotypes, and does not constitute an ideal taxonomic solution.

Ultimately, the present study supports the retention of these geotypes as distinct species. Although they likely represent lineages undergoing an incipient speciation process driven by recent geographic isolation, they remain distinguishable when evaluated through an integrative taxonomic framework that combines geographic distribution and molecular data.

The phylogenetic topology (Fig. 3) supports this evolutionary framework, resolving a highly supported monophyletic clade comprising the viperid-parasitizing species C. *bigenetica* and *C. jararacae* (posterior probability = 0.99; bootstrap = 100%). These results reinforce previous findings suggesting that *Caryospora* spp. underwent speciation as they became geographically isolated alongside their respective hosts in a host-parasite coevolutionary pattern, despite not maintaining a high level of strict host specificity [58, 59]. Although early-diverging reptilian lineages (including *I. amphiboluri*, *Lankesterella* sp., and *Caryospora* sp. from *P. major*) are positioned in a basal polytomy due to the current scarcity of multi-locus comparative data for reptilian coccidia, the target viperid *Caryospora* lineages consistently form a distinct and well-supported clade. As more *Caryospora* spp. from viperids are sequenced, phylogenetic analyses will become increasingly interpretive and conclusive. In particular, the inclusion of *C. simplex*, ideally from its type-host, will be essential to confirm and consolidate the observations presented in this study.

As shown in Table 1, in addition to the five species of the *C. simplex* species complex, two other morphologically distinguishable species are recorded from Viperidae. *Caryospora matatu* was described in detail by Modrý et al. [34]; meanwhile, *C. saudiarabiensis* was originally observed from *Echis coloratus* Günther, 1878 but misidentified by Alyousif et al. [35] as *Caryospora maxima* Modrý, Koudela, Al-Oran et Amr, 1999, a parasite recorded of Psammophiidae snakes. Subsequently, Duszynski & Upton [5] observed this same species parasitizing *Echis carinatus* (Schneider, 1801). Given the host specificity patterns at the family level documented among reptilian coccidia [2, 4–6, 15], and morphological similarities in their oocysts, Duszynski & Upton [5] established the name *C. saudiarabiensis* to correct this likely misidentification. Molecular sequencing of these two species will be highly elucidative, providing a baseline to measure genetic divergence among morphologically distinct viperid-infecting *Caryospora*. This will allow for a meaningful comparison with the subtle genotypic differences observed in the present study between the closely related species *C. bigenetica* and *C. jararacae*.

## Conclusions

In conclusion, through an integrative taxonomic approach to *Caryospora* spp. infecting Viperidae worldwide, *C. jararacae* is herein morphologically redescribed and molecularly characterized. The sequencing of mitochondrial and nuclear 18S rRNA loci provides the first genetically confirmed records for both *B. jararaca* and *C. durissus*, rendering *C. jararacae* only the second *Caryospora* species from a viperid host to be genotypically characterized globally. Furthermore, the *C. simplex* species complex is defined, comprising five geotypes distributed across different continents. Although these geotypes are morphologically indistinguishable, they can be delimited through an integrative framework combining biogeographical and genotypic data. Overall, the molecular data primarily support a scenario of recent common ancestry and incipient speciation, wherein high genetic similarity likely reflects shared ancestral characters following geographical separation, while potential gene flow facilitated by host dispersal remains a plausible secondary hypothesis to be tested in future population-level studies.

## Supplementary Information

Supplementary Material 1.

Supplementary Material 2.

## Data Availability

No datasets were generated or analysed during the current study.
